# Osteitis Condensans Ilii: A Case Series

**DOI:** 10.7759/cureus.28152

**Published:** 2022-08-18

**Authors:** Sreedhar Sathu, Maheshwar Lakkireddy, Ravi Kumar, Deepak Kumar Maley

**Affiliations:** 1 Department of Orthopaedics, All India Institute of Medical Sciences, Bibinagar, IND

**Keywords:** incidental, bone marrow edema syndrome, sacroiliitis, ankylosing spondylitis, osteitis condensans ilii

## Abstract

Osteitis condensans ilii (OCI) is a rare self-limiting low back pain syndrome. It is an incidental imaging discovery around sacroiliac joints with distinctive sclerotic lesions. We present three case reports as a series. In the first case, a 38-year-old female presented with unresolved chronic low back pain for eight years. She had bilateral triangular sclerosis of the ilium abutting sacroiliac joints and other causes of back pain were ruled out. Non-operative management was successful. In the second case, a 38-year-old female presented with acute exacerbation of low back pain for one year. She suffered low back pain during her pregnancy and postpartum period 16 years ago and intermittently after that. Bilateral radiodensity around sacroiliac joints was noted in the radiograph and she had successful remission with non-operative management. In the third case, a 45-year-old female presented with chronic low back for six years. On radiographs, she had bilateral sclerotic lesions around sacroiliac joints and responded well to non-operative management. OCI is a benign, idiopathic cause of axial low back pain. It is a diagnosis of exclusion and the pelvic radiographs classically show areas of sclerosis in the ilium adjacent to sacroiliac joints bilaterally. Treatment for OCI is essentially non-operative.

## Introduction

Osteitis condensans ilii (OCI) is a rare self-restricting, idiopathic situation marked using sclerosis of the iliac bone adjoining to the sacroiliac joints bilaterally. It is detected incidentally on pelvic imaging in otherwise asymptomatic individuals or ones suffering from low back pain [1]. OCI is often reported in females of childbearing age in pre or post-partum periods [2]. It is a benign pathological condition attributed to ligament laxity and mechanical stress across the sacroiliac joints with sclerosis typically limited to the iliac bone adjoining the sacroiliac joints [1]. In well-known, that it is an asymptomatic condition; however, in some patients, it manifests with lower back pain at a younger age similar to axial spondyloarthropathy (SpA) [3]. 

## Case presentation

Case 1

**Figure 1:**
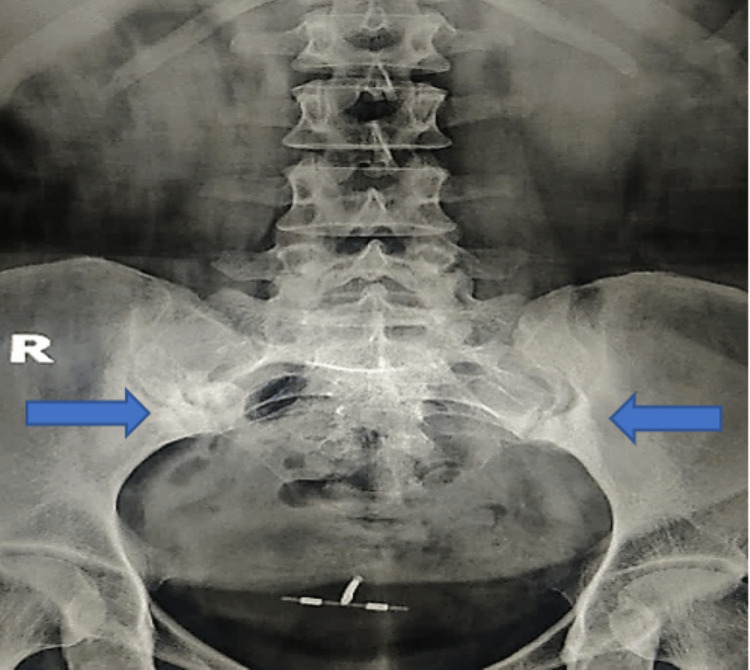
Antero-posterior view of the plain radiograph of pelvis showing triangular area of significant sclerosis over the inferior aspect of iliac border of both the sacroiliac joints.

A 38-year-old female presented to the orthopedic outpatient department (OPD) with a history of chronic low back pain ever since her first post-partum period (eight years). It continued through her second pregnancy (five years ago) and was severe as compared with the first episode. She had to undergo a cesarean section for both of her deliveries and there were no perioperative or postoperative complications. At present, she complains of low back pain in the lumbosacral area. It is intermittent in type and sharp in character. It gets aggravated by her routine household work, walking, lifting weights, and is relieved by rest. She has been using an intrauterine contraceptive device for the last five years. There is no history of morning stiffness, night cries, joint stiffness, swelling, deformity, dermatological, ophthalmological, bowel and bladder symptoms, weight loss or decreased appetite, or constitutional symptoms. Physical examination and straight leg raising test were normal. Flexion, abduction, and external rotation of the hip (FABER) did not elicit pain. Anteroposterior radiograph of the pelvis with both hips showed significant sclerosis at the iliac border of both the sacroiliac joints (Figure 1). Hematological investigations were normal (Table 1).

**Table 1:** Laboratory parameters of Case 1

Test	Results
Erythrocyte sedimentation rate	26 mm/hr (0-30 mm/hr)
C-reactive protein	Negative (< 6 mg/L)
Serum uric acid	4.5 mg/dl (3.5-7.2 mg/dl)
RA Factor	Positive (> 10 IU/ml)
Anti-cyclic citrullinated peptide (anti-CCP)	Negative (< 20 u/ml)
Anti-Nuclear Antibody (ANA) titre	Positive
Anti-Nuclear Antibody (ANA) profile	
nRNP/Sm	Negative
Sm	Negative
SS-A	Negative
Ro-52	Negative
SS-B	Negative
Scl-70	Negative
PM-SCL(PM)	Negative
Jo-1(ELISA)	Negative
CENP B	Negative
PCNA	Negative
dsDNA	Negative
Nucleosomes	Negative
Histones	Negative
Ribosomal-P Protein (PO)	Negative
AMA-M2(M2)	Negative

No other source of inflammatory pathology was identified. She was reassured of the condition and was advised non-steroidal anti-inflammatory drugs (NSAIDs) and physical therapy with advice to continue the exercises at home. Interferential therapy was used as a means of physiotherapy along with core strengthening exercises and pelvic exercises. She was comfortable after two weeks of treatment.

Case 2

**Figure 2:**
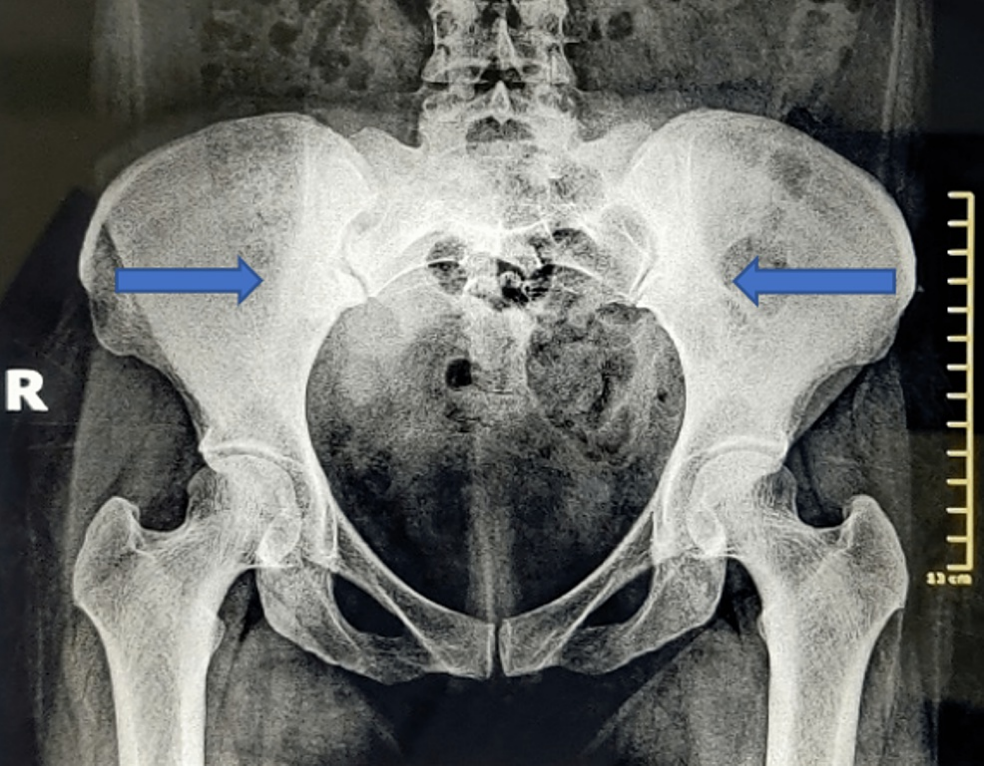
Antero-posterior radiograph of pelvis showing remarkable sclerosis at the iliac border of the bilateral sacroiliac joints.

A 38-year-old female patient presented to the orthopedic OPD with a history of localized and non-radiating low back pain over the lumbosacral area for one year. The pain was aggravated by forwarding bending and routine household activity and relieved by rest and analgesics. She suffered from low back ache during her pregnancy and the postpartum period around 16 years ago which continued intermittently with some symptom-free periods until a year ago when she developed severe back pain which persisted till date. Her pregnancies were four years apart and she had to undergo a cesarean section for both deliveries. There is no history of morning stiffness, night pain, or any other joint stiffness, swelling, deformity, dermatological, ophthalmological, bowel, and bladder symptoms. There is no history of weight loss or decreased appetite. General physical examination and straight leg raising test were routine. FABER did not elicit pain. A radiograph of the pelvis with both hips showed sclerosis at the iliac border of sacroiliac joints bilaterally (Figure 2). Other laboratory investigations were normal (Table 2).

**Table 2:** Laboratory parameters of Case 2

Test	Results
Haemoglobin	12.9 g/dl (12-16 g/dl)
Total white cell count (WBC)	10250 cells/cumm (4000-11000/cumm)
Erythrocyte sedimentation rate	30 mm/hr (0-30 mm/hr)
C-reactive protein	Negative (< 6 mg/L)
Rheumatoid factor	Negative (< 10 IU/ml)
Complete urine examination	Normal

No other possible source of inflammation was identified. She responded well to NSAIDs and physical therapy and continued the exercise program at home.

Case 3

**Figure 3:**
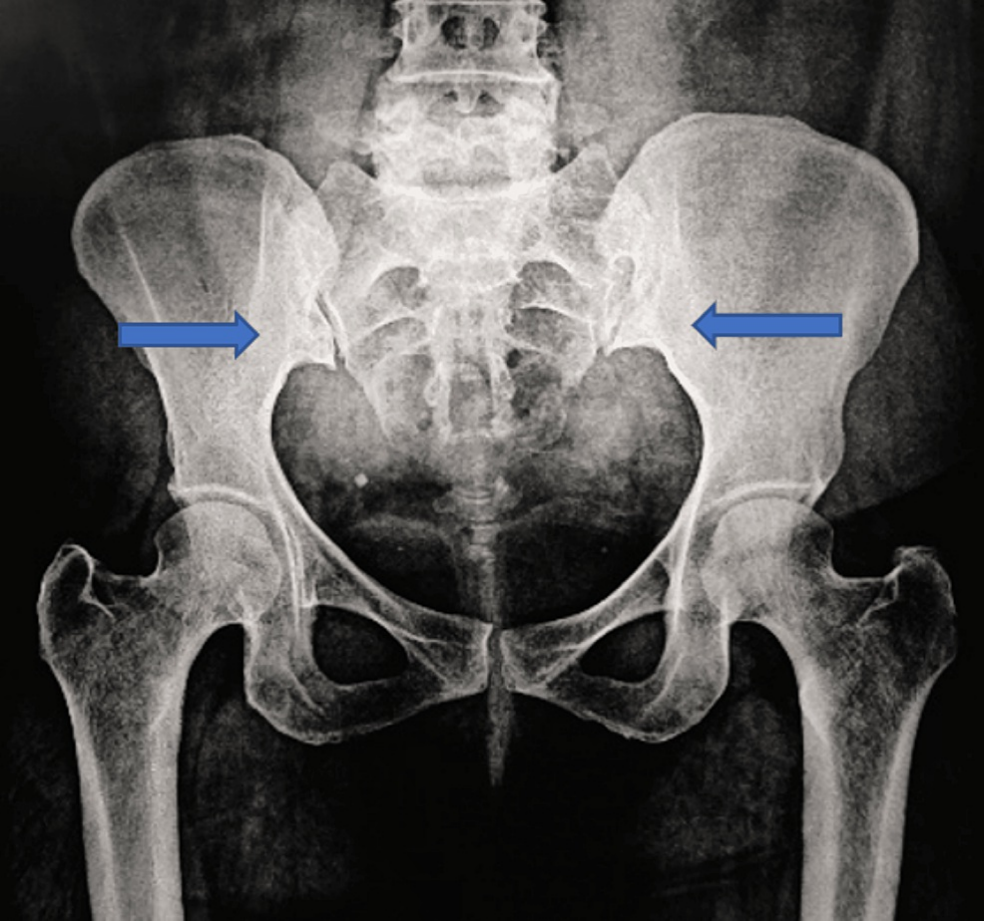
Antero-posterior radiograph of pelvis showing sclerosis at iliac border of sacroiliac joints.

A 45-year-old female presented to OPD with a history of chronic lower back pain for the last six years. She developed back pain during her second pregnancy (28 years ago) which continued in the postpartum period and subsided subsequently. She had severe low back ache in the third trimester of her third pregnancy and during her fourth pregnancy. It continued throughout the postpartum period and decreased later on. She had intermittent remissions and exacerbations on and off with milder intensity. She had to undergo a cesarean section for all the deliveries. For six years, the intensity of back pain was high over the lower lumbosacral area. It was aggravated by routine household activity, forward bending, lifting weights, and relieved by taking rest. There is no history of morning stiffness, night pain, joint stiffness, swelling, deformity, dermatological, ophthalmological, bowel, and bladder symptoms. There was no history of weight loss or decreased appetite. Physical examination, and straight leg raising test were routine and FABER was negative. A radiograph of the pelvis with both hips showed bilateral sclerosis at the iliac border of sacroiliac joints (Figure 3). Hematological investigations were normal (Table 3).

**Table 3:** Laboratory parameters of Case 3

Test	Results
Haemoglobin	10.8 g/dl (12-16 g/dl)
Total white cell count (WBC)	8750 cells/cumm (4000-11000/cumm)
Erythrocyte sedimentation rate	58 mm/hr (0-30 mm/hr)
C-reactive protein	Negative (< 6 mg/L)
Rheumatoid factor	Negative (< 10 IU/ml)
Serum uric acid	5.3 mg/dl (3.5-7.2 mg/dl)

No other source of inflammatory pathology could be identified. She had good relief with NSAIDs, physical therapy, and a continued exercise program at home.

## Discussion

Sicard, Gally, and Haguenau (1926) described cases with a condition now known as osteitis condensans ilii (OCI) for the first time. Bairsony and Polgair (1928) introduced the name of the disease for a specific clinical entity with the lesion found on radiological examination of the lumbosacral area with dense sclerosis in the iliac bone, adjacent to the lower part of the sacroiliac joint [4]. In general population estimation of prevalence is between 0.9 and 2.5% [5]. It is more common in young adults, with a predilection for females below 40 years of age, often following pregnancy [6-8]. Histopathologic findings are confined, it has been observed that the affected areas of sclerosis have focal bone marrow fibrosis, quantitative growth in lamellar bone, and accelerated trabecular density with the growth in osteoblastic activity [9]. Pain in OCI is commonly bilateral and may be radiating to the buttocks and posterior aspect of the thighs in a non-radicular type. Symptoms can also or won’t correspond with point tenderness on the sacroiliac joint. When deep pressure is applied posteriorly over the joint causes pain, Fortin's finger sign is positive. Flexion, abduction, and external rotation of the hip cause pain which is variably positive and similarly to nonspecific. Other provocative tests like sacral distraction tests, thigh/sacral thrust tests, and iliac compression tests are also unreliable measures.

While evaluating a patient with acute or chronic low back ache and suspicious of OCI radiologically, a detailed history and physical examination, including rectal (male and female) and gynaecological examination in female patients to rule out pelvic inflammatory disease and adnexal pathologies should be performed. Although OCI is localized to the lower back, it is essential to perform a full musculoskeletal examination to assess other joints. On physical examination, it is important to rule out congenital and post-traumatic causes, including leg-length discrepancy and spinal malalignment. Neurologic examination to find out any sensory or motor loss. Usually, no laboratory abnormality is seen in OCI. Laboratory investigations are most helpful to check the other causes of sacroiliac disease. Inflammatory markers like erythrocyte sedimentation rate (ESR) and C-reactive protein (CRP) are done to rule out an inflammatory arthropathy such as rheumatoid or psoriatic arthritis, or infectious sacroiliitis. HLA-B27 is rarely positive [10].

If the suspicion of the referred pain or adjacent internal source and inflammatory spondyloarthropathies are without history or supporting physical or laboratory findings then the attribution of symptoms to OCI would be appropriate. Sclerosis of the subcortical articular surface of the iliac bone extending into the adjacent medullary space, classical appearance as a triangular area of accelerated density/sclerosis with cephalad apex is the hallmark of OCI in radiography. Most commonly these radiographic findings are symmetrical. The vital discriminator within the radiographic findings is not related to any joint space narrowing or widening, ankylosis, erosion, effusion, bony destruction, bone marrow edema, or surrounding soft tissue edema. Advanced imaging techniques such as magnetic resonance imaging (MRI) and computed tomography (CT) might improve the ability to distinguish OCI from other conditions in cases of subtle findings or in cases where other differential diagnoses are considered to rule out any sclerotic conditions of the bone along with relevant clinical history, signs and symptoms. Otherwise, plain radiograph with distinct features matching the classical history and clinical examination findings are sufficient to diagnose OCI. More expensive imaging looking for early axial spondyloarthropathies can be reserved for those with signs and symptoms warranting higher investigations or who fail to respond to OCI therapeutic interventions. Even though isolated iliac involvement is a hallmark, it has also been shown that in a few cases, mild sacral sclerotic involvement is also seen [11].

The main goal of the treatment is to lessen the intensity and period of pain, and stiffness, and to enhance the patient's satisfaction and quality of life. The initial step in the management is the reassurance of the patient concerning the benign nature and the lack of progression of the disease clinically and radiographically. Because of the non-progressive nature of the disease, it is a very favorable prognosis, and the preferred method of treatment is conservative therapy. It comprises NSAIDs, rest, and physical therapy. Even though OCI is not an inflammatory condition, corticosteroid/anesthetic therapeutic injections have been employed and in one refractory case, a surgical core decompression was reported [12].

## Conclusions

OCI, although typically an incidental radiographic finding in asymptomatic patients, may be associated with lower back pain. In spite of the fact that no clear etiology has been identified, clinical history and triangular sclerosis of the iliac bone adjacent to sacro iliac joints are helpful in diagnosis. Sclerosis is confined to the ilium and may give the wrong impression of sacroiliac joint involvement. If strongly suspicious of other inflammatory conditions clinically, radiologically and serologically, advanced imaging techniques like MRI and CT scan would be helpful. Clinicians must recognize this disease entity and avert the use of potentially harmful and expensive regimens. Treatments for the condition are primarily non-operative (physical therapy, non-steroidal anti-inflammatory medications, rest as needed).
